# Meningococcal core and accessory phasomes vary by clonal complex

**DOI:** 10.1099/mgen.0.000367

**Published:** 2020-04-29

**Authors:** Joseph J. Wanford, Jonathan C. Holmes, Christopher D. Bayliss, Luke R. Green

**Affiliations:** ^1^​ Department of Genetics and Genome Biology, University of Leicester, Leicester, UK; ^2^​ Department of Infection, Immunity and Cardiovascular Disease, University of Sheffield, Sheffield, UK

**Keywords:** clonal complex, carriage, hypervirulence, microevolution, *Neisseria*, phase variation

## Abstract

*
Neisseria meningitidis* is a Gram-negative human commensal pathogen, with extensive phenotypic plasticity afforded by phase-variable (PV) gene expression. Phase variation is a stochastic switch in gene expression from an ON to an OFF state, mediated by localized hypermutation of simple sequence repeats (SSRs). Circulating *N. meningitidis* clones vary in propensity to cause disease, with some clonal complexes (ccs) classified as hypervirulent and others as carriage-associated. We examined the PV gene repertoires, or phasome, of these lineages in order to determine whether phase variation contributes to disease propensity. We analysed 3328 genomes representative of nine circulating meningococcal ccs with Phasome*It*, a tool that identifies PV genes by the presence of SSRs and homologous gene clusters. The presence, absence and functions of all identified PV gene clusters were confirmed by annotation or blast searches within the *Neisseria* PubMLST database. While no significant differences were detected in the number of PV genes or the core, conserved phasome content between hypervirulent and carriage lineages, individual ccs exhibited major variations in PV gene numbers. Phylogenetic clusters produced by phasome or core genome analyses were similar, indicating co-evolution of PV genes with the core genome. While conservation of PV clusters is high, with 76 % present in all meningococcal isolates, maintenance of an SSR is variable, ranging from conserved in all isolates to present only in a single cc, indicating differing evolutionary trajectories for each lineage. Diverse functional groups of PV genes were present across the meningococcal lineages; however, the majority directly or indirectly influence bacterial surface antigens and could impact on future vaccine development. Finally, we observe that meningococci have open pan phasomes, indicating ongoing evolution of PV gene content and a significant potential for adaptive changes in this clinically relevant genus.

## Data Summary

Phasome*It* analyses are included as a supplementary dataset on Zenodo (DOI: 10.5281/zenodo.3545849) (https://zenodo.org/record/3545849#.XoxM_HJKiM8). An Excel file is included [MGL Library (11.12.2018).xlsx] that describes the available isolates when the Meningococcal Genome Library (MGL) was interrogated. Further worksheets are provided to demonstrate which isolates were included for each clonal complex (cc) and the random choice of isolates. Compressed files of each Phasome*It* analysis grouped by cc are included, within these folders are three .html files; index.html, which summarizes all of the Phasome*It* analysis; core_phasome.html, describes the core phasome for the cc; tractLengths.html, describing each phase-variable gene tract length. Three folders are included that make up the data files for the described .html files.

Supplementary Material is available with the online version of this article.

**Impact Statement**

Phase variation is a high frequency mutational mechanism by which bacteria switch expression of a gene ON or OFF, which allows them to rapidly adapt to new environments, such as upon transmission to a new host. The ability to phase vary genes can be detected from genome sequences by identifying the presence of simple sequence repeats in the ORF. In the UK, genomes from clinical isolates of the major human pathogen *Neisseria meningitidis* are routinely sequenced and deposited in the open PubMLST database. In this study, we performed a comparative analysis of the presence of phase-variable (PV) genes in 3328 genomes encompassing major carriage-associated and hypervirulent meningococcal clones. Whilst we detected no discernible difference in the number or functions of PV genes between carriage and hypervirulent clones, we found divergent repertoires of PV genes between related groups that may shed light on the selection pressures driving continual evolution of these clinically relevant clonal complexes. We anticipate our data will form a framework for understanding the implications of phase variation on carriage dynamics, and transmission throughout the population.

## Introduction

*Neisseria meningitidis* (the meningococcus) is a Gram-negative, obligate human commensal and pathogen. Normally, meningococci are found as asymptomatic colonizers of the human nasopharynx, but they can occasionally cross the mucosal barrier to cause septicaemia and meningitis [1]. However, the switch from carriage to an invasive state remains poorly understood, and whilst genomic correlates can be found with disease-causing potential, these do not explain the dual behaviour of this pathogen. One potential determinant of the disease to carriage switch is localized hypermutation and the consequent phase-variable (PV) expression of key surface molecules.

Phase variation is a high-frequency switch in gene expression, often conferred by localized hypermutation at simple sequence repeats (SSRs) in the genome [2]. Addition or deletion of repeats by slippage of the replicative polymerase at these loci can confer changes in gene expression from an ON to an OFF state through inducing frameshifts (if the SSR is within an ORF) [2], or can alter levels of gene expression between low, intermediate and high states (if the SSR is in a promoter or regulatory region) [3]. Importantly, PV expansion/retraction of SSRs occurs at a frequency vastly greater (>1×10^−5^ mutations per division) than macro-evolutionary events in the genome, such as SNPs or movement of transposable elements [4, 5]. Characterization of SSRs in genome sequences allows assessment of the propensity of a particular clone to vary surface phenotypes by phase variation. Furthermore, the protein products of many PV genes are outer-membrane proteins (OMPs), and selection for ON/OFF phase variation in these loci are often driven by recurrent host selection through bactericidal antibodies [6, 7]. Initial studies of meningococcal genome sequences identified ~50 PV genes with the potential to generate large amounts of genetic and phenotypic heterogeneity in otherwise isogenic bacterial populations [8]. Recently Aidley *et al.* [9] developed Phasome*It*, a new program for analysing whole-genome sequences (WGSs) for the presence of PV genes subject to SSR-mediated switches in gene expression. Wanford *et al*. utilized this program to analyse SSR-PV genes across the genus *Neisseria* and observed that there were significant differences in the number of PV genes between *Neisseria* spp., with the majority of commensal species having low numbers of PV genes, whilst *Neisseria lactamica* and the pathogenic *Neisseria* had ~40–60 PV genes per genome [10]. An intra-species analysis of phasome content across clonal complexes (ccs), which could specifically identify differential PV content across distinct lineages of meningococci known to differ in their disease-causing potential, has yet to be undertaken.

Protection against invasive meningococcal disease (IMD) is achieved through multiple vaccines, both polysaccharide [11, 12] or protein-based [13], which elicit immunity against a subset of serogroups and lineages. Despite the efficacy of these vaccines, the circulation of non-vaccine-covered strains, serotype replacement and mutation-mediated vaccine escape remain a concern for public health [14, 15]. Vaccine design and clinical epidemiology have benefited from routine whole-genome sequencing of meningococcal disease isolates by allowing for detailed interrogation of the genomic population structure of circulating clones [16, 17]. The deposition of this genome data into the *Neisseria* PubMLST database [18] has provided an excellent resource for both clinical epidemiologists, and research into disease-associated genetic determinants [19]. Currently in the UK, most cases of disease are due to five major circulating ccs: cc11, cc213, cc269, cc41/44 and cc32. These lineages are a subset of the hypervirulent lineages that tend to have high disease to carriage ratios. Other ccs, such as cc22, cc23, cc162 and cc461, are infrequent causes of IMD and have low disease to carriage ratios; hence, they are referred to as carriage-associated. These ccs collectively account for 87 % of strains within the Meningitis Research Foundation (MRF) Meningococcal Genome Library (MGL) in *Neisseria* PubMLST. The WGS data deposited within the MGL provides an opportunity to interrogate whether phase variation influences how different meningococcal clones colonize their hosts and cause disease.

In this study, we have defined the collection of PV genes, the phasome, across nine currently circulating meningococcal ccs in the UK, using the novel tool Phasome*It* [9] to provide a conceptual framework for understanding colonization dynamics and pathogenesis. We hypothesize that differences in the phasomes between major ccs of meningococci have arisen due to specific variations in the within-host selection pressures experienced by these clones, such as the immune response to surface OMPs, and that these differences may have implications for rational vaccine design and evaluation.

## Methods

### Genomic dataset

Genome contigs were extracted from the MRF MGL available through *Neisseria* PubMLST [18] in December 2018. From among the 3568 WGSs available, we selected the ccs represented by >1.5 % of isolates (i.e. >54) for analysis (Supplementary Material, available with the online version of this article). This dataset covered the following ccs: cc11 (*n*=815), cc162 (*n*=60), cc213 (*n*=224), cc22 (*n*=79), cc23 (*n*=499), cc269 (*n*=529), cc32 (*n*=165), cc41/44 (*n*=669) and cc461 (*n*=75). A subset of 10.1 % of the isolates did not have a defined cc, these genomes were analysed using the PubMLST tool ‘Search by combination of alleles’ to identify partial multilocus sequence typing (MLST) profiles allowing for assignment to relevant ccs. The remaining 213 isolates were classified as ‘new ST’ (new sequence type). Contig files were converted to annotated GenBank files using prokka (v. 1.11) [20] with MC58 as a reference sequence. All genome sequences and their respective metadata are shown in the Supplementary Material. All Phasome*It* runs included a number of reference strains, such that loci could be rapidly identified by their reference strain number in the PubMLST allele database. These strains were: MC58, H44/76, NZ-05/33, α710, FAM18, M25419, DE10444, M01-240355 and M04-240196.

### Extraction of PV gene repertoire using Phasome*It*

Phasome*It* was run as described elsewhere [4, 5, 10, 21, 22] for each cc individually (containing 75–815 genomes per cc). An additional run was performed combining a random selection of 50 genomes from each cc. The cut-offs for the repeat numbers for each repeat tract type were as follows: 9 for poly-G repeat tracts, 11 for poly-T tracts, 8 for dinucleotide repeat tracts, 6 for trinucleotide tracts, 5 for tetranucleotide and pentanucleotide tracts, and 3 for hexanucleotide to nonanucleotide tracts. These cut-offs were developed based on available data highlighting the propensity of these SSRs to undergo indels for *Neisseria* [4, 5, 10, 21, 22]. The BioPython module Bio.Phylo was used to reconstruct neighbour-joining trees indicating the separation of isolates using a distance matrix that was derived from the Manhattan distance metric from binary lists of presence/absence of gene groups.

### Downstream core-phasome analysis using Phasome*It* data

Core phasomes were derived for each cc individually using Phasome*It.* PV genes were considered core if they were present and contained an SSR in more than 50 % of isolates. To facilitate comparison across different cc datasets, Phasome*It* functional groups were assigned NEIS numbers by querying the reference strain gene numbers against the allele definitions provided by the *Neisseria* PubMLST database; thus, grouping together all alleles of a given loci. Where a gene identified in the analysis was not present in any of the reference strains, the first identified sequence of this loci in Phasome*It* was directly queried against the PubMLST allele database. Core-phasome genes for each cc were subsequently cross-checked across all other cc datasets, to identify the percentage PV conservation of these loci. This comparative approach was required, as if a locus is PV in one cc but not in another, then it is considered part of the accessory phasome. The analysis pipeline used in this work is outlined in Fig. S1.

### Presence/absence gene analysis

Phasome*It* will only identify a locus if a PV tract is present within one of the isolates from a cc [9, 10]. As each cc was analysed individually, it is possible that non-PV loci were not identified during the initial inquiry. Therefore, the compiled list of NEIS numbers corresponding to all core and accessory PV genes were queried against all of the strains previously analysed using the ‘Gene Presence’ function in PubMLST. Gene Presence utilizes a blastn query (v1.4.16) [23] with 70 % identity and 50 % coverage to produce a ‘% present’ matrix for all genes across each cc. To further confirm the presence or absence of loci of interest, reference sequences of each loci were queried using blastn with default parameters against a 50 genome subset from each cc within the PubMLST database. The two matrices, (i) % present in the phasome taken from Phasome*It*, and (ii) % present across the cc taken from the Gene Presence analysis, formed the basis of subsequent analysis.

### Additional routine bioinformatic analysis

To confirm the blast-based sequence alignments employed by Phasome*It* were identifying multiple alleles of the same gene, and not loci with substantial regions of homology (e.g. comparison of *porA* and *porB* sequences), we interrogated a number of loci using the ClustalOmega sequence alignment tool (v1.2.4) [24]. Loci of interest were extracted from a subset of 25 genomes and aligned using ClustalOmega with default parameters. Alignments were analysed to confirm the repeat number described by Phasome*It.* Localization of SSRs and their context within the genome were visually interrogated using Artemis genome visualization software (v17.0.1) [25].

### Statistical analysis

Statistical analysis of the numbers of PV genes in each cc was performed using a two-way ANOVA in GraphPad Prism software.

## Results

The genus *Neisseria* exhibits significant variation in the numbers of PV genes, for example, *Neisseria flavescens* has three PV genes per genome, while meningococci have a mean of 39 per genome [10]. This species-to-species variation indicates that the number of PV genes may be influenced by niche specificity or alternate biological behaviours. As meningococcal ccs differ in their propensity to persist in carriers and to cause disease, we speculated that PV repertoires may vary between ccs. In order to test this hypothesis, we examined the PV signatures of nine ccs that were split into hypervirulent (cc11, cc41/44, cc213, cc32, cc269) [26] or carriage-associated/endemic (cc22, cc461, cc23, cc162) lineages [27]. The non-groupable isolates, not associated with a cc and identified as a new ST, were excluded from this classification system.

### Detection and clustering of PV genes in different meningococcal ccs

**Fig. 1.**
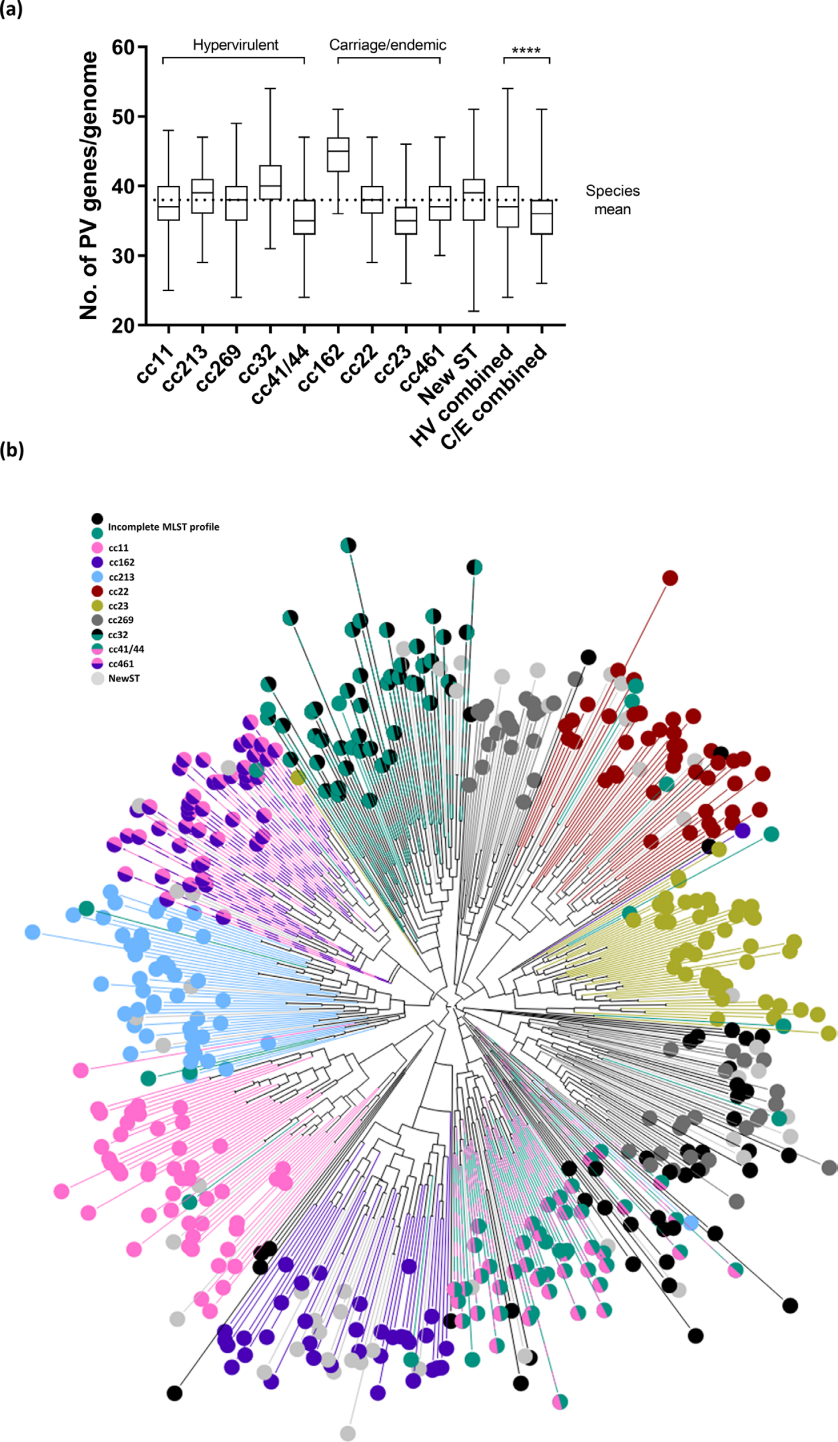
Clustering of PV genes in meningococcal ccs. (a) Box plot depicting the range, median, and first and third quartiles of the number of PV genes detected for each cc. Statistical significance was analysed by two-way ANOVA. ****, *P* <0.0005. The mean number of PV genes across all ccs is depicted with a dotted line. C/E, Carriage/endemic; HV, hypervirulent. (b) Neighbour-joining tree based on the presence or absence of PV homology groups across all ccs. Colours corresponding to each cc are shown in the key.

Phasome*It* identifies PV genes in genome sequences based on the presence or absence of an SSR. The PV gene content was delineated for each of the major UK circulating ccs and a set of non-groupable strains (the new ST group) by grouping SSR-containing genes and non-PV homologues based on a 50 % sequence homology/coverage cut-off. We observed that each cc contained between 22 and 55 PV genes (Fig. 1a). The mean number of PV genes per genome was significantly lower in typically carriage-associated ccs compared to hypervirulent ccs (35 and 37 PV genes, respectively; *P* <0.0001, two-way ANOVA). Of the hypervirulent ccs, cc32 encoded the largest number of PV genes (40.5±4.1) when compared to cc11 (37.6±3.8), cc41/44 (35.6±3.9), cc213 (38.3±3.8) and cc269 (37.6±3.8). Of the typically carriage/endemic ccs, cc162 encoded the greatest mean number of PV genes (44.4±3.4) compared with cc22 (37.9±3.5), cc461 (37.7±3.5) and cc23 (34.7±3.1). Whilst strains designated new ST displayed an analogous number of PV genes (40) to other ccs, they had the largest range of detected PV genes (22–50). Statistical comparisons of the number of PV genes in each cc is detailed in the Table S1.

Phylogenetic relationships strongly influence gene content. To determine the similarities between the phasomes of the ccs*,* a neighbour-joining phylogenetic tree was derived based on the presence or absence of PV genes. ccs form discreet clusters that are delineated by specific PV gene content (Fig. 1b). Isolates of the new ST group exhibited ‘mixed clustering’ with respect to their PV gene content, suggesting that these isolates are closely related to the known lineages, but the association is masked by divergent or incomplete MLST profiles. In the majority of cases, clustering by phasome reflected the relatedness of meningococcal isolates by MLST and whole-genome phylogenetic analysis. For example, cc162 and cc269 share a root on the phasome neighbour-joining tree, and this mimics the relatedness of these isolates by MLST [28], in which these isolates are more closely related to each other than to other ccs. In contrast, cc213 and cc32 appear highly related on a phylogenetic tree based on the core genome (Fig. S2), but are separated on the phasome tree. These data indicate that PV gene repertoire mimics genomic phylogeny, but that there is a mobile gene pool with the potential to move PV genes between clonally unrelated ccs.

Repetitive DNA can cause problems in genome assembly, such as contig breaks, leading to highly fragmented genome assemblies [29]. Identification of functional gene groups by Phasome*It* relies on the ability to assign an SSR to an ORF [9]. To infer whether the differences detected in PV gene content between ccs was due to actual gene content or due to missing loci in the genome assembly, we analysed the correlation between the number of contigs in each cc data set (Fig. S3a) versus the number of PV genes detected by Phasome*It* (Fig. S3b). In agreement with previous work [10], we detected a negative correlation whereby the number of PV genes decreased as the number of contigs increased, which was significant for every cc except cc162 (Fig. S3b). This negative correlation is probably due to a small number of PV genes with longer repeat tracts that cause contig breaks and, hence, they are not detected by Phasome*It*. In general, the low correlation suggests there are minimal undetectable repeat tracts; therefore, they do not affect the conclusions of this work.

### ccs contain a core phasome alongside a distinctive accessory phasome

**Fig. 2.**
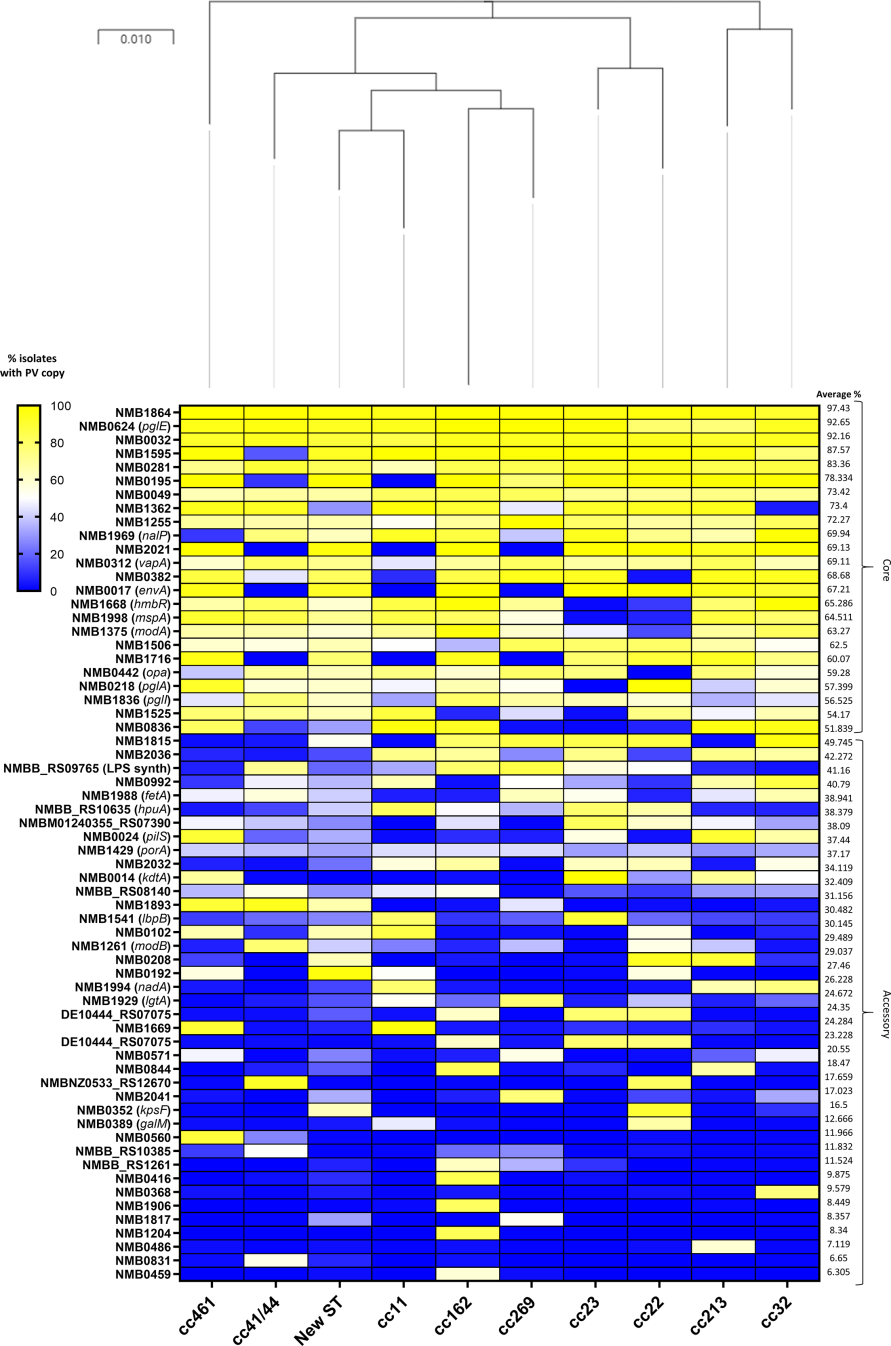
Presence of PV genes across each cc. Core-phasome data were used to derive the per cent presence of PV genes above a 50 % threshold for all isolates. Where genes were core in one cc, but not in another, the respective loci were queried against the ‘non-core’ cc dataset to provide the per cent conservation below the 50 % core-phasome cut-off. Genes are sorted from top to bottom, on the highest mean conservation of phase variation across all ccs. The colour gradient represents the percentage of isolates with a PV copy of the respective gene ranging from blue (low conservation) to yellow (high conservation). To give an overview of population structure, an MLST tree was included based on the PubMLST typing scheme for meningococcus using one representative isolate from each cc. The MLST tree was drawn using iTOL software, through the PubMLST interface where the scale bar denotes single nucleotide polymorphisms.

To determine the specific differences in PV gene content driving the Phasome*It* clustering of ccs, we performed a comparative analysis of the percentage of isolates in each cc that contained specific ‘core phasome’ PV genes (Fig. 2). We found that 24 PV gene families were present in greater than 50 % of genomes across all ccs, these genes are representative of the species core phasome as reported by Wanford *et al*. [10]. However, only three genes (NMB1864, *pglE* and NMB0032) were present in the core phasomes of all ccs (Fig. 2). Presence of a gene in the core phasome of a cc tended to show a binary pattern, whereby a gene was either present or absent from all isolates with very few genes showing an intermediate presence in the phasome. An exception was NMB1255, which had a 50 % presence in the cc11 phasome. The core phasomes of each cc showed a variable amount of overlap, with 43 genes in three or more ccs, 7 genes in two, and 13 genes only in a single cc (e.g. NMB0416 is present in 100 % of cc162 isolates, but not in any isolates of the other ccs). Lastly, the presence of specific genes in the phasome did not necessarily reflect the relatedness of isolates by MLST analysis. For example, cc213 and cc32 share a common root on the MLST tree, but have different core phasomes with 80–100 % variation in the presence of several genes (Fig. 2).

Phasome*It* analysis includes non-PV homologues of PV genes identified through a networking approach (see the work by Aidley *et al.* [9]). However, Phasome*It* may not identify a specific PV gene in a cc if there are no PV homologues of that gene in any of the strains of that cc. To circumvent this issue, we utilized core-phasome locus tags (i.e. NEIS numbers) to query the PubMLST database and generate a presence/absence analysis for each cc irrespective of whether that gene contained an SSR (see the heat map in Fig. S4). This analysis indicated that despite sparse conservation of genes in the phasome (Fig. 2), 48 out of the 63 core-phasome genes were present in all isolates of all nine ccs. This indicates that there is variable conservation between ccs of the SSR within these 48 genes and not loss of these genes.

**Fig. 3.**
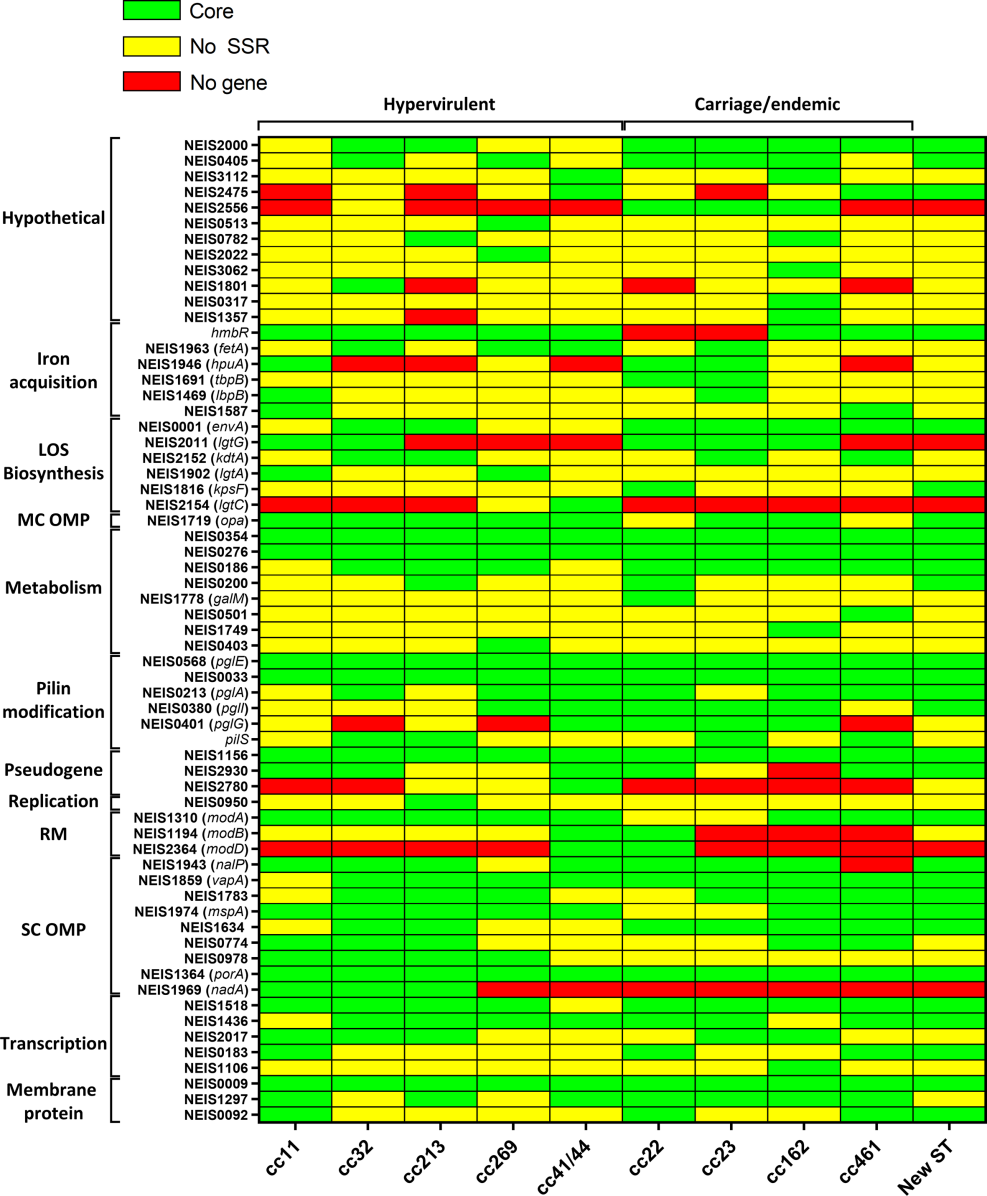
Presence of modular PV genes and their cognate SSRs in hypervirulent and carriage-associated meningococci. The heatmap demonstrates whether genes were in the core phasome. Bars are depicted as follows: core – present in greater than 50 % of isolates and phase variation in greater than 50 % of isolates (green); no gene – absent from the core because of lack of the loci in 50 % of isolates (red); or no SSR – presence of the gene but lacked an SSR in 50 % of the isolates (yellow). Strains are grouped based on their predicted virulence (hypervirulent vs carriage), genes are arranged into modules by their known or predicted function. LOS, Lipo-oligosaccharide; MC, multi-copy; OMP, outer-membrane protein; RM, restriction modification; SC, single-copy.

To examine the distribution of PV genes in more detail, we coded the PubMLST presence/absence data, and the per cent presence Phasome*It* data (Fig. 3) to produce a binary matrix, outlining whether a gene was present in the core phasome (>50 % PubMLST; >50 % Phasome*It*), present but without an SSR (>50 % present in PubMLST; <50 % Phasome*It*) or not present (<50 % PubMLST; <50 % Phasome*It*), as a proxy for the origin of PV loci in these ccs. Interestingly, the majority of gene groupings across all ccs were not present in the core phasome due to lack of presence of an SSR, with the remaining minority non-core-phasome genes lacking the gene altogether. Detailed analysis of conservation of specific functional groupings of genes will be described below.

### Restriction modification systems

Consistent with previous work [30], our analysis identified PV copies of the three restriction modification systems encoded by the *modA*, *modB* and *modD* loci. The PV *mod* alleles were detected by the presence of five or more repeats and were associated with GCCA, CCCAA and ACCGA repeats (Table 1). *modA* was highly conserved, being present in all ccs, and was subject to phase variation in all ccs except cc22 and cc23 (Fig. 3), which contained an SSR in less than 50 % of isolates. *modB* was less conserved, being absent from cc461, cc162 and cc23 (Fig. 3). Where present, *modB* was subject to phase variation in only cc41/44 and cc22. Finally, *modD* was only present in cc41/44 and cc22, and was also PV in these ccs (Fig. 3).

**Table 1.** Functional analysis of core and accessory PV genes of meningococcus Functions of genes were either discerned from published literature where available or from predicted functional domains using a blastp search. Predominant SSRs are those that make up more than 50 % of the loci across the dataset. Evidence for phase variation was split into the following: known, functional studies in the literature; alignment, SSR present and differential tract length observed at homologous loci; and SSR present, SSR identified in the ORF but no alignment-based evidence for phase variation.

NEIS number (gene)	Function/functional domain	Predominant SSR	ccs gene conserved and subject to phase variation	Evidence for variation
**LOS biosynthesis**				
NEIS0001 (*envA*)	UDP-3-*O*-acyl *N*-acetylglucosamine deacetylase	GC	32, 213, 22, 23, 162, 461, new ST	SSR present
NEIS2011 (*lgtG*)	LOS glucosyltransferase	C	11, 32, 22, 23, 162	Known [6]
NEIS2152 (*kdtA*)	Addition of Kdo moiety to lipid A	G	32, 213, 23, 461	SSR present
NEIS1902 (*lgtA*)	*N*-Acetylglucosaminyltransferase	C	11, 269	Known [59]
NEIS1816 (*kpsF*)	Rabinose-5-phosphate isomerase	GC	22, new ST	SSR present
NEIS2154 (*lgtC*)	Galactosyltransferase	G	41/44	Known [59]
**Pilin modification**				
NEIS0568 (*pglE*)	Glycosylation of pilus	CAACAAA	All	Known [60]
NEIS0033 (*pilC2*)	Retraction of pilus	C	All	Known [61]
NEIS0213 (*pglA*)	Glycosylation of pilus	G	32, 269, 41/44, 22, 162, 461, new ST	Known [60]
NEIS0380 (*pglI*)	Glycosylation of pilus	G	269, 41/44, 22, 162, new ST	Known [60]
NEIS0401 (*pglG*)	Glycosylation of pilus	C	41/44, 22, 23, 162	Known [60]
*pilS*	Silent loci which recombines with *pilE*	GCCGAC	32, 213, 23, 461	SSR present
**Restriction modification**				
NEIS1310 (*modA*)	Methylation and foreign DNA restriction	GCCA	11, 32, 213, 269, 41/44, 162, 461, new ST	Known [30]
NEIS1194 (*modB*)	Methylation and foreign DNA restriction	CCCAA	41/44, 22	Known [30]
NEIS2364 (*modD*)	Methylation and foreign DNA restriction	ACCGA	41/44, 22	Known [30]
**Single-copy OMPs**				
NEIS1943 (*nalP*)	Autotransporter	C	11, 32, 213, 41/44, 22, 23, 162, new ST	Known [62]
NEIS1859 (*vapA*)	Contains autotransporter domain (UniProt)	GCAA	32, 213, 269, 41/44, 22, 23, 162, 461, new ST	SSR present
NEIS1783	Outer membrane protein class 4	GAACCC	32, 213, 269, 23, 162, 461, new ST	SSR present
NEIS1974 (*mspA*)	Autotransporter	C	11, 32, 213, 269, 41/44, 162, 461, new ST	Known [62]
NEIS1634	Membrane fusion protein/efflux pump	GC	32, 213, 22, 23, 162, 461, new ST	SSR present
NEIS0774 (*clpA*)	ATP-dependent Clp protease	TGAAGA	11, 32, 213, 162, 461	SSR present
NEIS0978	Adhesin	C	11, 32, 213, 269	SSR present
NEIS1364 (*porA*)	Porin	G	All	Known [63]
NEIS1969 (*nadA*)	Adhesin/invasin	TAAA	11, 32, 213	Known [3]
**Iron acquisition**				
hmbR (*hmbR*)	Haemoglobin receptor	G	11, 32, 213, 269, 41/44, 162, 461, new ST	Known [64]
NEIS1963 (*fetA*)	Ferric enterobactin transporter	C	32, 269, 41/44, 23	Known [65]
NEIS1946 (*hpuA*)	Haemoglobin receptor	G	11, 22, 23	Known [66]
NEIS1691 (*tbpB*)	Transferrin-binding protein	ATAACAAA	22, 23	SSR present
NEIS1469 (*lbpB*)	Lactoferrin receptor	AAGCTG	11, 23	SSR present
NEIS1587 (*pigA*)	Haem utilization protein (haem oxygenase)	GAAGCC	11, 461	SSR present
**Metabolism**				
NEIS0354 (*hemL*)	Glutamate-1-semialdehyde aminotransferase	CGGTTG	All	SSR present
NEIS0276	Peptidyl-prolyl cis-trans isomerase	GCCAAAGCT	All	SSR present
NEIS0186 (*pdxA*)	4-Hydroxythreonine-4-phosphate dehydrogenase	GC	32, 213, 269, 22, 23, 162, 461, new ST	SSR present
NEIS0200	Ferredoxin	GC	213, 22, new ST	SSR present
NEIS1778 (*galM*)	Aldose 1-epimerase; carbohydrate metabolism	CCGCTACCC	22	SSR present
NEIS0501	Serine acetyltransferase	CCGCGG	461	SSR present
NEIS1749	UDP-MurNAc-pentapeptide synthetase	CGC	162	SSR present
NEIS0403 (*ribD*)	Riboflavin biosynthesis protein RibD	CGG	269	SSR present
**Transcription/translation**				
NEIS1518 (*alaS*)	Alanyl-tRNA synthetase	ACGCGC	11, 32, 213, 269, 22, 23, 162, 461, new ST	SSR present
NEIS1436 (*argS*)	Arginyl-tRNA synthetase	ACGCGC	32, 213, 269, 41/44, 22, 23, 461, new ST	SSR present
NEIS2017 (*truA*)	tRNA pseudouridine synthase A	C	11, 32, 213, 23, 162	SSR present
NEIS0183	Ribonuclease HII	T	11, 22, 461, new ST	SSR present
NEIS1106	Transcriptional regulator	TG	162	SSR present
**Hypothetical**				
NEIS2000	na	CG	32, 213, 22, 23, 162, 461, new ST	SSR present
NEIS0405	na	GCCAAAGCT	32, 269, 22, 23, 162, new ST	SSR present
NEIS3112	na	CAAG	41/44, 162	SSR present
NEIS2475	na	CCTT	41/44, 461, new ST	SSR present
NEIS2556	na	AT	22, 23, 162	SSR present
NEIS0513	na	G	269	SSR present
NEIS0782	na	ACGGAT	213, 162	SSR present
NEIS2022	na	CCTGTTT	269	SSR present
NEIS3062	na	ATTATC	162	SSR present
NEIS1801	na	A	32	SSR present
NEIS0317	na	GACACG	162	SSR present
NEIS1357	na	C	162	SSR present
**Multi-copy OMP**				
*opa*	Adhesion to epithelial cells	CTTCT	na	Known [45]
**Pseudogene**				
NEIS1156	Homology to *lgtA*	G	All	SSR present
NEIS2930 (*icsA*)	Autotransporter precursor	AGCA	11, 32, 41/44, 22, 461, new ST	SSR present
NEIS2780	Type-1 restriction enzyme specificity MPN_089	G	41/44	SSR present
**Other**				
NEIS0950	Replication initiation	G	213	SSR present
NEIS0009	Uncharacterized membrane protein	A	All	SSR present
NEIS1297	Uncharacterized membrane protein	TAGGCT	11, 213, 41/44, 22, 23, 162, 461	SSR present
NEIS0092	Uncharacterized membrane protein	G	11, 22, 461, new ST	SSR present

### Pilin glycosylation

Four pilin glycosylation loci, *pglA, pglI, pglE* and *pglG* [31], were identified as being associated with G, G, CAACAAA and C repeats, respectively (Table 1). The most conserved phase-variation-associated *pgl* gene was *pglE,* which was both present and highly prevalent as a PV locus across all ccs (92 % phase-variation conservation across all genomes). Similarly, *pglI and pglA* were present in >50 % of isolates across all ccs, but were not subject to phase variation in three and four ccs, respectively (*pglA* – cc11, cc23, cc213; *pglI* – cc461, cc11, cc213, cc32). *pglG* was absent from three ccs (cc461, cc269 and cc32) and only subject to phase variation in cc41/44, cc162, cc23 and cc22 (Fig. 3). Of note, we did not identify *pglH* in our phase-variation analysis, indicating that any repeat tract present in this gene either fell below our SSR cut-off or was missed in the fragmented assembly (see below).

### Lipo-oligosaccharide (LOS) biosynthesis

PV LOS modifying genes, *lgtG*, *lgtA* and *lgtC* [32], were identified in association with tracts of nine or more G, G and C repeats, respectively (Table 1). *lgtA* was the only one of these three genes to be present in all ccs, but was only PV in two hypervirulent clones, cc11 and cc269 (Fig. 3). *lgtC* was not present in any of the carriage/endemic-associated ccs but was present in two hypervirulent clones, cc269 and cc41/44, and only PV in the latter cc. *lgtG* was not present in the carriage-associated ccs cc461 and the new ST, nor in the hypervirulent clones cc213, cc269 and cc41/44 (Fig. 3). In the remaining ccs, this gene was present and subject to phase variation. For some isolates, in the gene grouping encompassing *lgtG*, we observed a second homologous loci, although none of these were subject to phase variation. This can be due to other genes in the locus with high levels of homology to *lgtG*. We found no evidence of grouping of loci for the other *lgt* genes. In addition, we identified three novel PV loci with roles in synthesis/modification of the LOS. *kdtA* is responsible for addition of a Kdo moiety to lipid A in the early stages of LOS biosynthesis [33]. This gene was present in all ccs, and contained an in-frame PV poly-G SSR at the 3′ end of the gene, with a tract length range of 9–10 repeats (Table 1) in two hypervirulent ccs (cc32 and cc213) and two carriage-associated clones (cc23 and cc461).

We also observed that *envA* can be subject to phase variation due to a GC repeat tract. This SSR was present in all carriage-associated ccs, but only in cc32 and cc213 hypervirulent clones (Fig. 3). This gene encodes an UDP-3-*O*-acyl *N*-acetylglucosamine deacetylase that is putatively involved in LOS biosynthesis (Table 1) [34]. Another ubiquitously distributed gene, *kpsF,* encoding a rabinose-5-phosphate isomerase [35], was also found to contain a GC tract above the eight repeat unit cut-off. A PV version of *kpsF* was present in some isolates of cc22, and the new ST isolates (Fig. 3). A key future test is to demonstrate phase variation of one of these two genes, as dinucleotide tracts are not known to mediate phase variation in *Neisseria *.

### Iron acquisition

We identified the three known PV iron-acquisition systems, *hmbR, fetA* and *hpuA* [36], which were associated with polyG, C and G repeats (Table 1), respectively. A PV copy of *hmbR* was absent from the carriage-associated cc22 and cc23 lineages, but present in >50 % of isolates across all other ccs (Fig. 3). The *fetA* gene was present in all ccs; however, only showed >50 % phase variation within cc32, cc269, cc41/44 and the carriage-associated cc23 (Fig. 3). Similarly, the *hpuA* gene was present within two hypervirulent lineages (cc11 and cc269) and three carriage-associated lineages (cc162, cc22 and cc23), but only generally PV within cc11, cc22 and cc23 (Fig. 3).

In an extension of this module, SSR tracts were identified in three iron-acquisition genes not typically considered as subject to phase variation; *tbpB* (encoding transferrin-binding protein) [37], *lbpB* (encoding lactoferrin-binding protein) [38] and *pigA* (a *Pseudomonas* homologue involved in haem acquisition) [39] (Table 1). These genes were associated with ATAACAAA, AAGCTG and GAAGCC repeats with phase-variation thresholds of six repeats. The former of these tracts would be subject to ON/OFF phase variation, whereas the latter two would be subject to in-frame phase variation. All ccs encoded *tbpB*, but only the carriage-associated cc22 and cc23 lineages encoded PV copies in >50 % of isolates (Fig. 3). Similarly, *lbpB and* NEIS1587 (*pigA*) were ubiquitous, but only contained PV SSRs in hypervirulent cc11 and the carriage-associated cc23 isolates (Fig. 3).

### Other OMPs

Our results also confirm and extend observations on the distributions of several other PV single copy OMPs [40–45]. Both the *porA and nadA* genes were always present as PV loci, with the former present in all ccs (Fig. 3), and the latter only in the cc11, cc32 and cc213 hypervirulent lineages. Analyses of the two PV autotransporters indicated that *mspA* was universally present in all lineages, but only contained SSRs in hypervirulent lineages and two carriage-associated lineages, cc162 and cc461 (Fig. 3). Similarly, *nalP* was encoded by all hypervirulent and carriage-associated ccs except cc461, and was PV in all except cc269 (Fig. 3).

Due to the homology cut-off employed for generating homology groups in Phasome*It*, we were unable to separate highly homologous, multi-copy genes into individual loci as expected for the *pilC1/pilC2* and the four *opa* loci. Analysis of the numbers of *opa* loci (Fig. S5) indicates that the modal number of *opa* detected across all ccs was three, with 1–3 PV copies per genome. However, the modal number of *pilC* loci detected was 2–3 per genome. This is contrary to the expected two loci. Resolution of multi-copy PV genes is likely to improve with the availability of higher-quality genome sequences. Values for these loci are, therefore, representative of the percentage of PV versus non-PV for all these loci grouped together. We found that all ccs have at least one PV copy of both the *opa* and *pilC* loci.

### Exemplars for the absence of phase variation: short SSRs, interrupted SSRs and problems in genome assembly

Lack of an SSR in a widely distributed PV gene can be either of evolutionary importance or due to errors in assembly of these repetitive sequences. Absences of SSRs in key loci were investigated by aligning gene sequences across multiple isolates with differing propensities for phase variation. Alignment of *fetA* sequences of representative isolates from the cc11 (PV) and cc32 (non-PV) lineages revealed that non-PV tracts had an SSR of length C6, which is below the threshold for a PV gene (Fig. S6a). Contrastingly, alignment of the *kdtA* sequences of a representative non-PV isolate, from cc11, and a PV isolate, from cc32, revealed a polyG tract in the non-PV isolate that was interrupted by a C nucleotide substitution (Fig. S6b). The *kdtA* gene is organized as part of a putative operon with NMB0013 and NMB0012 downstream (Fig. S6c). While translation of *kdtA* with a G10 tract is predicted to yield three distinct protein products, deletion of a single G nucleotide from the SSR results in a longer protein product due to fusion of the first two ORFs (Fig. S6d, e). This gene was shown to be non-essential but required for optimal fitness, as knockout *kdtA* mutants have impaired growth in rich media, with longer logarithmic doubling times and a lower maximum OD_600_ [33].

An unexpected result was a lack of phase variation in some of the *pgl* genes. Aligned sequences of *pglA, pglI* and *pglH* were generated for ccs where these genes are either present or absent from the core phasomes. The findings were mixed, with *pglA* losing the propensity for phase variation due to interruption of the SSR (Fig. S7a), while *pglI* was missing from the list of PV genes due to difficulties in genome assembly producing contig breaks within the SSR (Fig. S7b). The *pglH* gene was absent due to either highly dissimilar repeat regions or contig breaks (Fig. S7c). Resolution of these assembly problems and improved SSR detection could be achieved by the use of long-read sequencing technologies to supplement draft genomes produced by the Illumina platform.

### Comparison of core- and accessory-phasome size between carriage and virulent strains

**Fig. 4.**
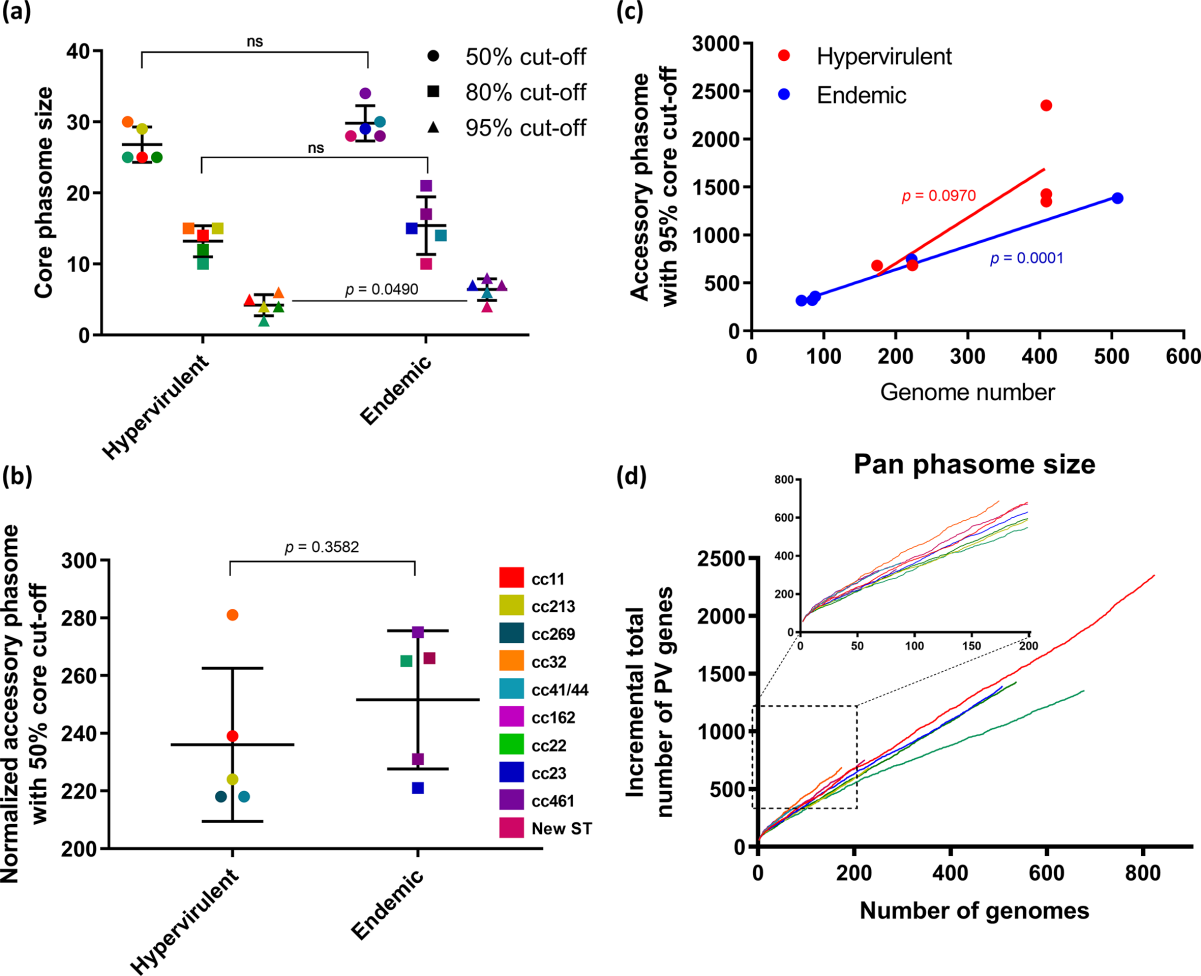
Core- and accessory-phasome sizes of meningococcal ccs. (a) The size of core phasome was derived for each cc, by setting a cut-off of being present and PV in greater than 50, 80 or 95 % of isolates. Statistical significance was determined by t-test, ns denotes no significance. (b) Accessory-phasome sizes of each cc derived from a random sample of 50 genomes from each cc. Statistical significance was determined using a *t*-test. (c) Correlation between total number of genome sequences analysed for each cc and the size of the accessory phasome. Statistical significance was determined using Pearson‘s correlation. (d) Pan-phasome analysis of the number of PV genes detected with an incremental number of genomes analysed for each cc. The inset shows the range where genomes were still being added for all ccs. Data points in (a), (b) and (d) corresponding to different ccs are colour coded as displayed in the key.

Incidences of IMD are thought to occur soon after strain acquisition, as the carriage state elicits a protective immune response and may also result in adaptation to the nasopharyngeal niche, driving meningococci into a non-invasive state. Differences in the ability to generate genetic and phenotypic diversity, therefore, may play a role in the propensity for strains to cause disease following recent host colonization. Therefore, we examined whether differential core PV gene repertoires could be observed between typically carriage-associated or endemic meningococcal ccs, and hypervirulent lineages. Fig. 4(a) indicates the size of core phasomes for each cc for three different cut-offs for conservation of phase variation: 95, 80 and 50 %. With the most stringent cut-offs, we found that the hypervirulent meningococci had smaller core phasomes than carriage-associated lineages (*P*<0.05). This significant difference is due to the loss of PV genes and/or absence of PV genes of a small gene set in the hypervirulent lineages that disappears at lower cut-offs as these missing genes merge into larger PV-containing gene homology groups. We interpret this modest difference as indicating that the core phasome is required for establishing a carriage state without impacting on the virulence potential of this organism.

The accessory-phasome sizes were also examined for each meningococcal cc as a measure of the variability in phasomes. We defined the accessory phasome as gene groupings absent from the core phasome. A count of the accessory phasome was performed independently for each complete cc dataset with a 50 % cut-off. No significant differences in the accessory-phasome size were detected between meningococcal ccs suggesting that phase-variation capacity is similar across all lineages of this genus. Due to the differing number of strains in each cc, we analysed a random sample of 50 genomes from each cc. In this case, a large degree of variation was observed in the accessory-phasome size between ccs; however, carriage and disease-associated ccs were not significantly divergent (Fig. 4b). The cc with the largest accessory phasome was cc32, which had an accessory PV gene pool of ~250 deriving from 162 genomes, and a core-phasome size of only 28 genes. To further assess the accessory phasome, we compared the number of genomes present in a cc with the number of accessory PV loci (Fig. 4c). There was a significant correlation between genome number and accessory-phasome sizes for all combined ccs and for carriage-associated strains, but not for hypervirulent strains. As the number of PV genes has expanded with analysis of additional genomes, we examined whether the meningococcus has an open or closed pan phasome. We observed a trend for continual detection of PV genes (open pan phasome), but with differences between lineages such that cc11 trends towards more PV genes than cc41/44, indicative of a higher degree of genomic plasticity (Fig. 4d).

## Discussion

Hypervirulent and carriage-associated meningococcal lineages have differing propensities to cause disease that are likely to have a genetic component [24]. Our analyses observed a modest difference in the combined frequency of PV genes between typically hypervirulent and carriage-associated ccs, but with no clear pattern of specific genes differentiating these phasomes. This indicates that PV gene content is not a determinant of hypervirulence, despite experimental evidence for many of these genes contributing to experimental invasive disease [46, 47]. Only *nadA* exhibited a bias towards hypervirulent lineages, being present in 3/5 of these lineages but absent from the carriage-associated lineages. Conversely, the majority of PV loci were present in both hypervirulent and carriage-associated ccs, indicating that PV gene content is a major determinant of carriage dynamics not disease propensity. This finding places the onus on analysis of the expression state of PV genes when considering whether these genes contribute to hypervirulence.

Whilst case studies of longitudinal carriage are readily available in the literature [16, 48], there is limited information on the lengths of carriage specific to particular lineages. We can speculate that different ccs may be predisposed to different periods and rates of carriage. Phase variation is considered an adaptive mechanism to reduce the fitness costs imposed by transmission bottlenecks, which may facilitate survival in a new host in the face of strong selective pressures by host immunity [49]. In this scenario, the number of PV genes encoded by a cc is a proxy for the ability to adapt to new environments with a consequent influence on lengths and rates of carriage. Interestingly, we observed that cc162 has a significantly larger phasome size than all other ccs leading to the prediction that this cc will have a higher propensity for persistent carriage than other lineages. The influence of PV gene content on carriage dynamics will require further investigation.

Typing schemes based on the genome sequence of bacterial pathogens have allowed for the accurate tracing of population evolution [50].WGS phylogenetics of meningococci indicate the presence of clades containing ~2 ccs and numerous STs [51]. The clades and ccs have fairly deep evolutionary roots. We show that ccs cluster phylogenetically by their phasome content, indicating that co-evolution of the core genome and the phasome may be a common feature of all meningococcal lineages. However, there are examples of clustering of closely related ccs in the phasome analysis. This observation implies ongoing expansion/retraction of repeat tracts mediated by a diverse set of selection pressures that is independent of the core genome.

The only vaccines licensed for targeting protection against serogroup B meningococci are the protein-based Bexsero and Trumenba [13]. These vaccines are not fully effective against all MenB strains, whilst the emergence of vaccine escape strains and serotype replacement remain important concerns. Evaluation of the conservation of surface-exposed structures and their propensity to antigenically vary is required to develop novel broadly protective vaccines [52]. Our workflow will facilitate characterization of the likelihood of phase or antigenic variation of surface-exposed structures and whether there will be variation between ccs in these processes. For example, LOS is considered a vaccine target for gonococcus [53] and has previously been considered for the meningococcus [54], as in the face of potential vaccine escape, it may be a useful broadly protective antigen in the future [55]. Antibodies targeted against LOS core structures have been shown to be bactericidal for both species [56, 57]. Unexpectedly, our analysis revealed a varied pattern for putative phase variation of KdtA, with potential consequences for phase variation of LOS core structures across all lineages. These differential phase-variation capabilities may be a critical consideration when utilizing a specific LOS epitope as a vaccine antigen.

Within-host selection – such as exposure to antibodies – occurs with expression of surface-exposed structures. Modelling approaches have indicated that in the absence of selection, populations would drift towards loss of SSRs [58]. Despite this, SSRs are prevalent in a variety of bacterial pathogens [22]. In addition, the repeat number directly correlates with the mutability of the locus, with high numbers predisposing to higher mutation rates [4, 5]. Differences in SSRs – either absolute repeat number or the binary below/above threshold for mutability – are likely reflective of the particular set of selection pressures imposed upon a clonal lineage. Our analysis indicated that while the majority of PV genes are conserved in their presence/absence, there are discreet differences in the presence or absence of repeat tracts at these loci. These data provide a conceptual framework to understand how selection pressures shape the evolution of diverse meningococcal lineages. Furthermore, these data allow analysis of the mechanistic basis for the genesis of PV genes in this species, such as the expansion of a pre-existing SSR or recombination resulting in transfer of an SSR between strains.

## Data Bibliography

1. Wanford JW, Holmes JC, Bayliss CD, Green LR. Data deposited at Zenodo, DOI:10.5281/zenodo.3545849 (2019).

## Supplementary Data

Supplementary material 1

Supplementary material 2

Supplementary material 3
