# Supplementary material for: Meningococcal core and accessory phasomes vary by clonal complex
Source: Microb Genom. 2020 Apr 29;6(5):e000367. doi: 10.1099/mgen.0.000367 (PMC7371114; doi:10.1099/mgen.0.000367)
Supplement: Supplementary material 2 [file mgen-6-367-s002.pdf]

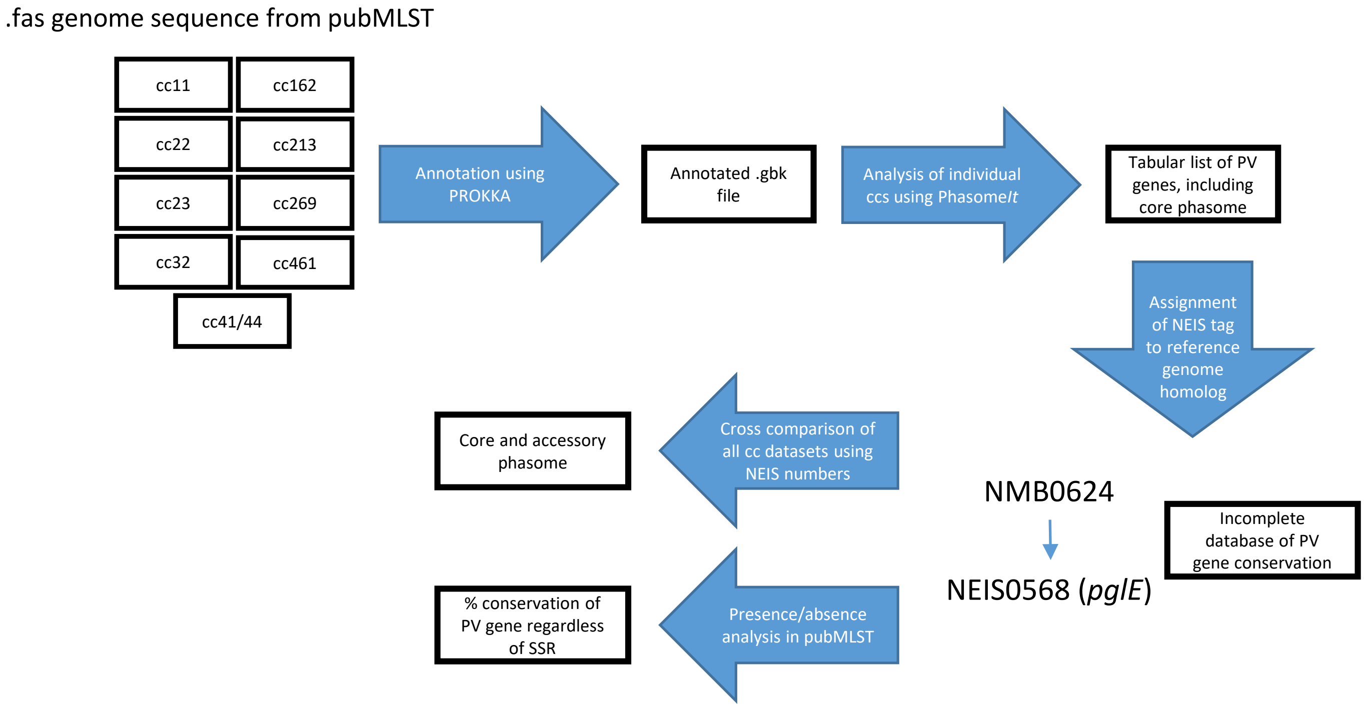

Supplementary Figure 1. Workflow for comparative analysis of core and accessory PV loci. Genome sequences are annotated with PROKKA using MC58 as a reference. Annotated genbank (.gbk) files are then split by clonal complex and analysed using Phasome*It*. The Phasome*It* output includes a tabular list of gene groupings, and the conservation of their PV status for all clonal complexes. To facilitate cross comparison of datasets, gene groupings are assigned NEIS numbers using the pubMLST allele designations. The complete set of identified alleles are required across all ccs to identify the PV conservation of sporadic genes across all datasets. In parallel, NEIS numbers are queried using the presence/absence function of pubMLST for all isolates to give a presence/absence matrix that can be used to interpret the Phasome*It* output. In the diagram, file outputs are shown in black boxes, whereas functions and analyses are shown in blue arrows.

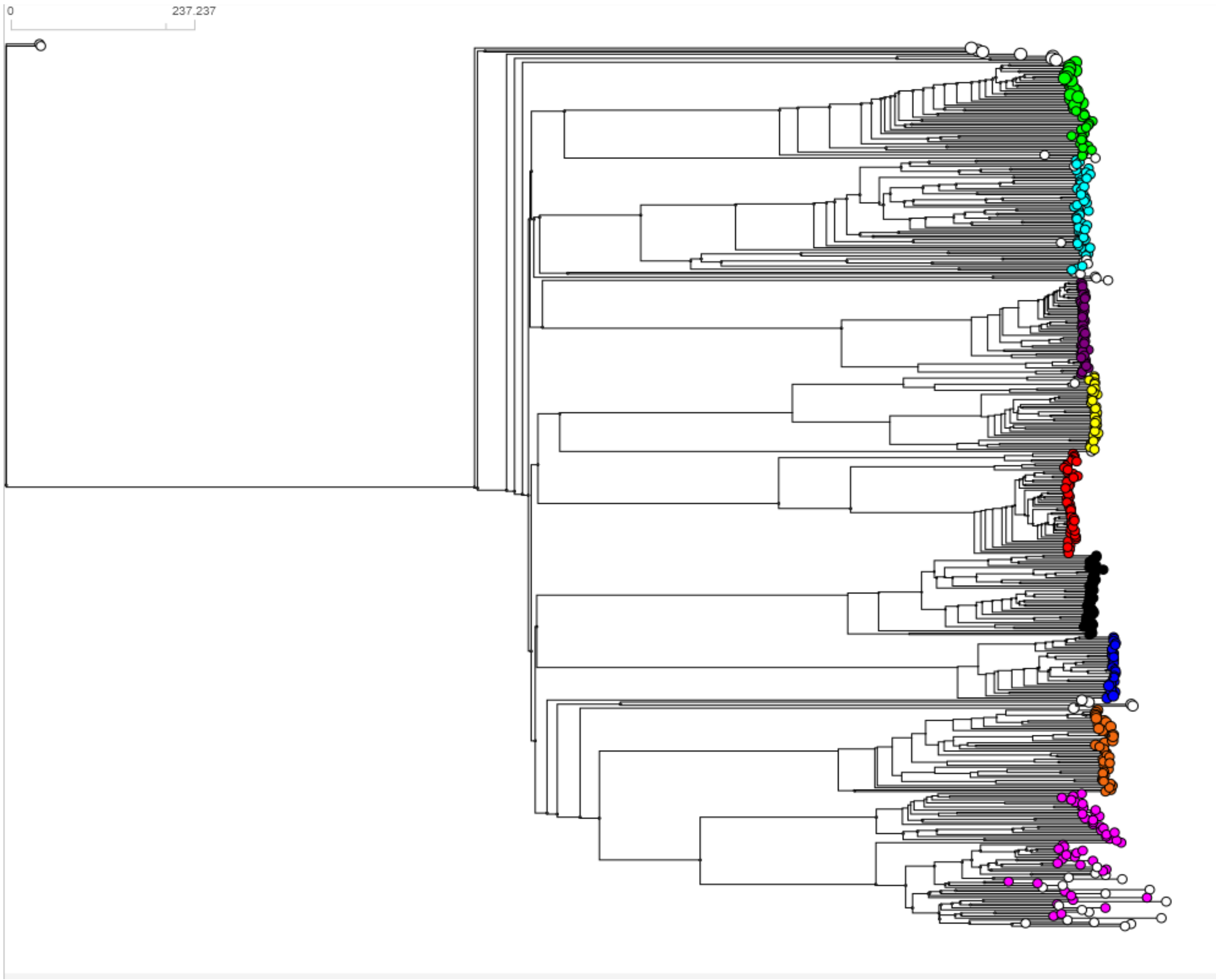

Supplementary Figure 2. Core genome phylogeny of representative strains from the ccs analysed in this study. A random sample of 50 isolates was selected from each cc, and a core genome comparison was performed with a minimum identity and alignment of 80% excluding incomplete loci using the pubMLST genome comparison tool. The tree was drawn with Splitstree5 using the neighbour joining algorithm. Pink; cc269, orange; cc32, black; cc461, cyan; cc41/44, purple; cc23, yellow; cc22, red; cc11, white; new ST, green; cc213, blue; cc162.

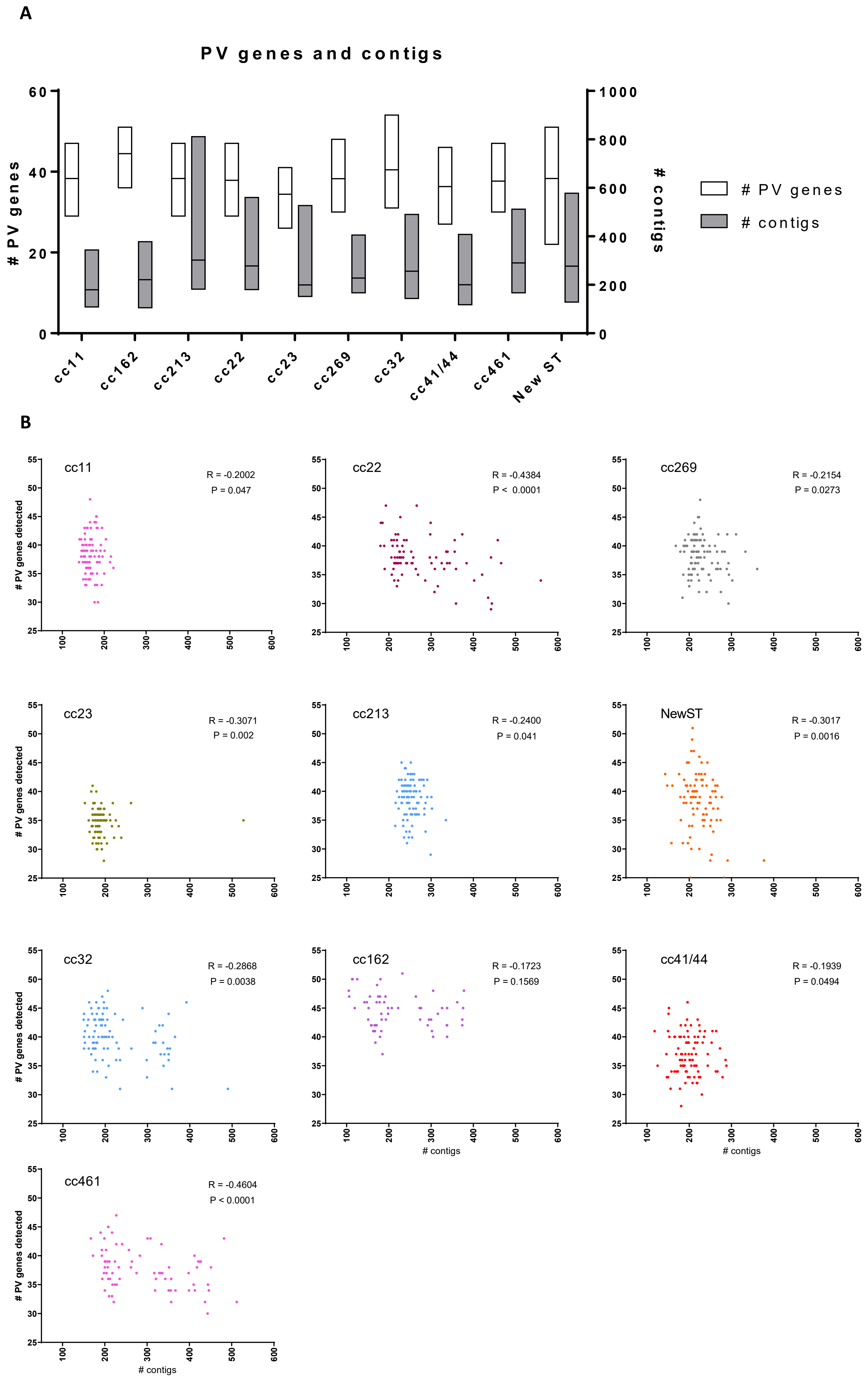

Supplementary Figure 3. Association of PV gene detection with fragmentation of genome sequence. (A) box plot displaying a comparison of the number of PV genes detected in all genomes from each CC, with the number of contigs the cognate genome is assembled into. (B) correlation plot of number of PV genes detected in each cc, with number of contigs making up the genome sequence. Correlations and statistical significance was assessed by Spearman.

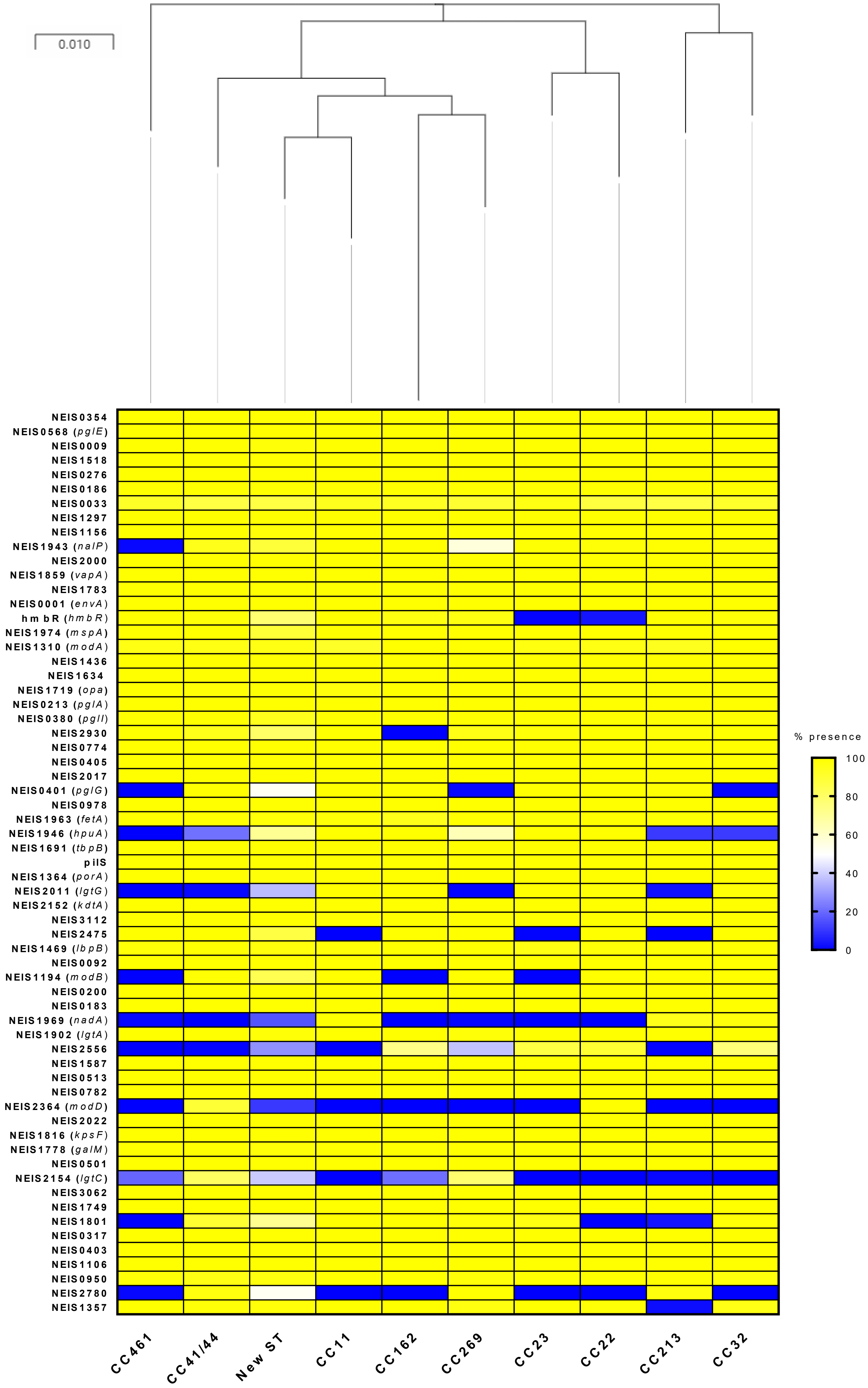

Supplementary Figure 4. Percentage presence/absence heatmap for genes identified in the core phasome analysis. NEIS numbers were queried against the genome sequences for all isolates of a given cc using the presence/absence function in the pubMLST *Neisseria* database. The colour gradient represents the % of isolates with a copy of the respective gene ranging from blue (present in 0% of isolates), to yellow (present in 100% of isolates). The phylogenetic tree is the same as shown in Figure 2.

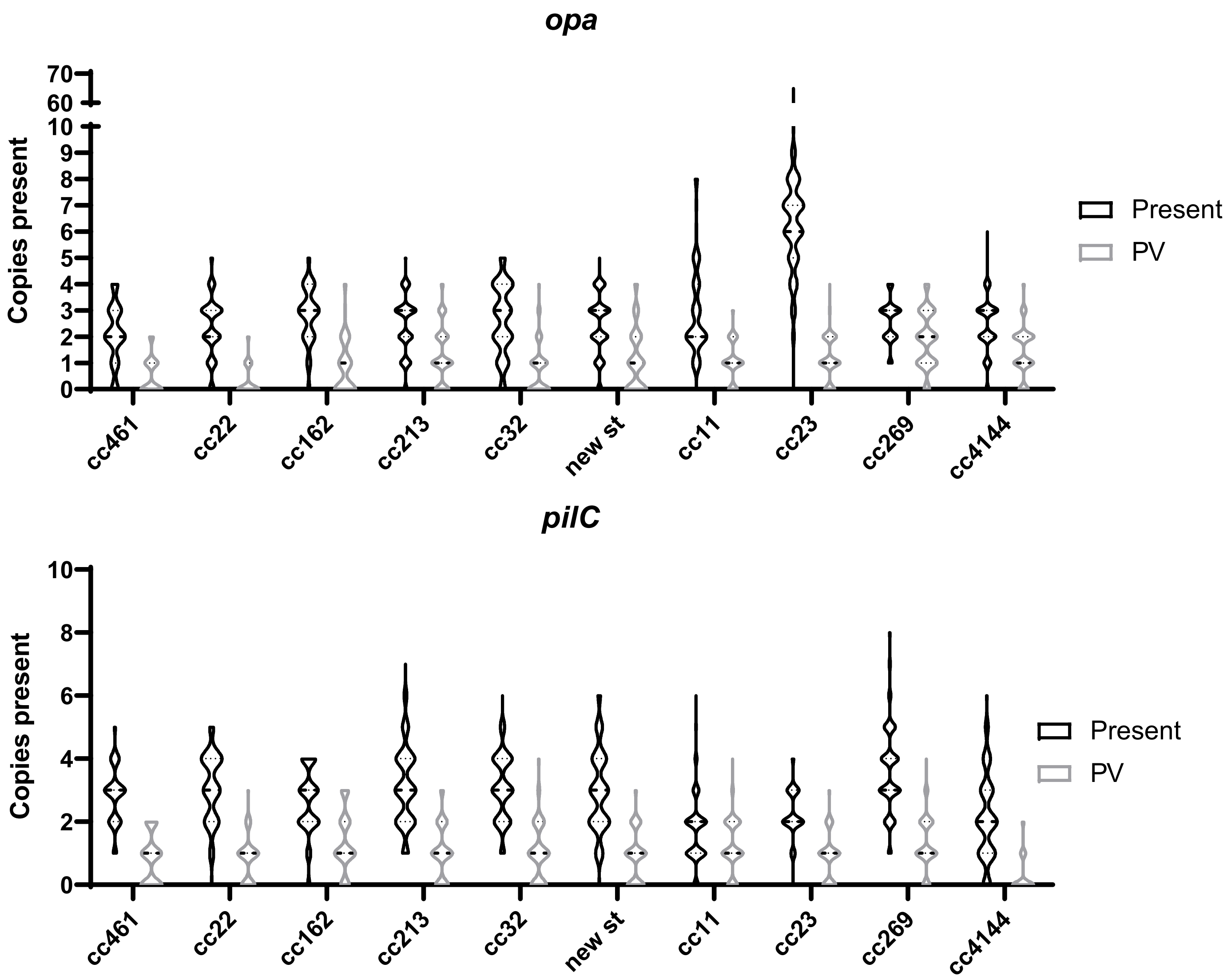

Supplementary Figure 5. Detection of multi-copy PV genes by Phasome*It*. Violin plot showing the number of loci detected by Phasome*It* (black; present) and the number of those which were subject to phase variation (grey; PV) in gene families for *opa* (A) and *pilC* (B). The frequency of loci is indicated by the width of the bar, whilst the mean is indicated by a dotted line within the bar.

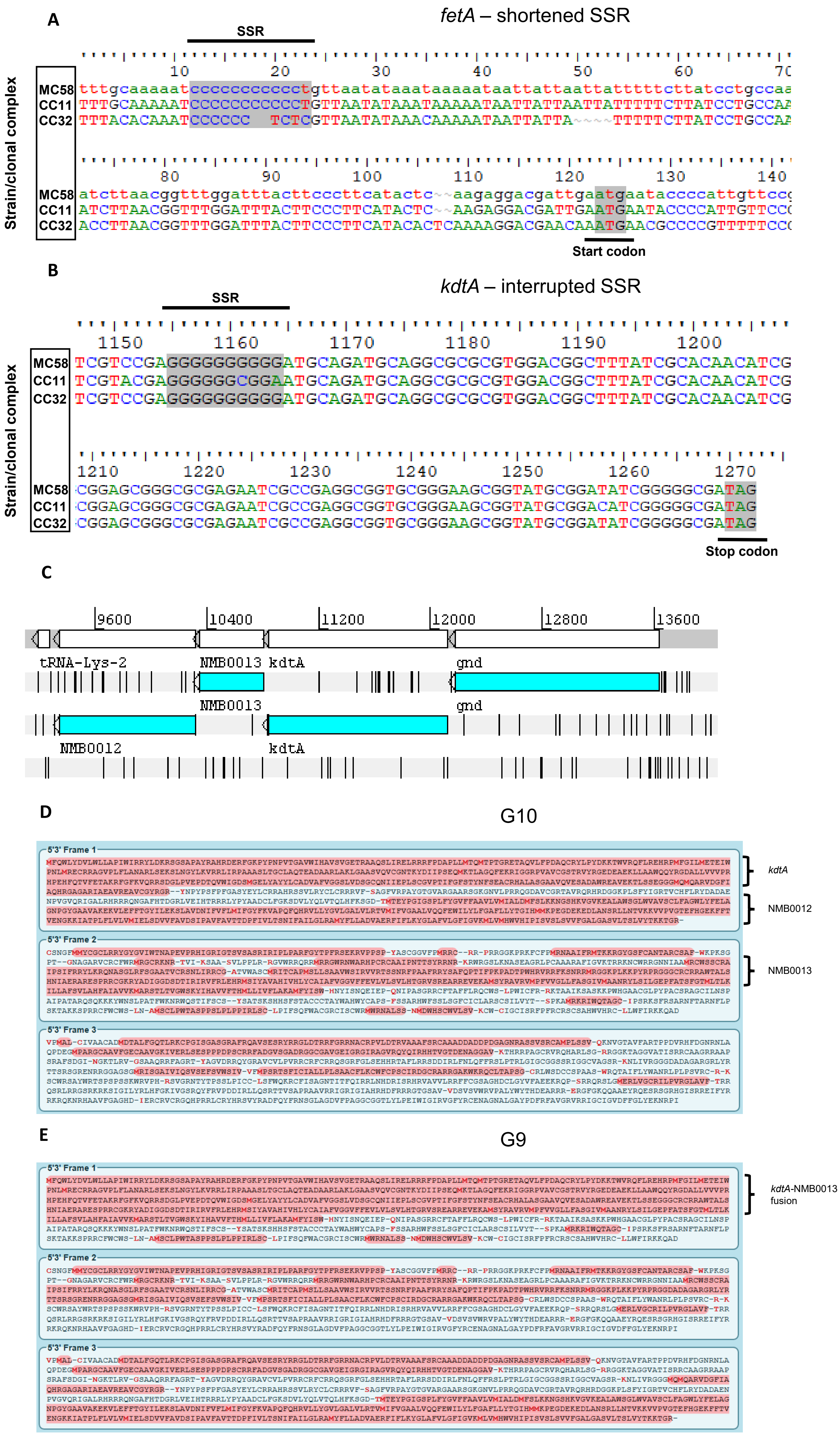

Supplementary Figure 6. Sequence alignments indicating the origin of SSRs in the exemplar genes *fetA* and *kdtA*. (A) nucleotide sequence alignment of the upstream region of the *fetA* gene including the SSR from reference strain MC58, a cc11 strain, and a cc32 strain. (B) nucleotide sequence alignment of the 3' region of the *kdtA* gene including the SSR from reference strain MC58, the cc11 strain, and the cc32 strain. (C) genomic context of the *kdtA* operon in reference strain MC58 visualised using the artemis genome comparator tool. (D) translation of the *kdtA* operon with the native G10 SSR of *kdtA* showing all 3 reading frames. (E) translation of the *kdtA* operon with an artificial deletion forming a G9 tract.

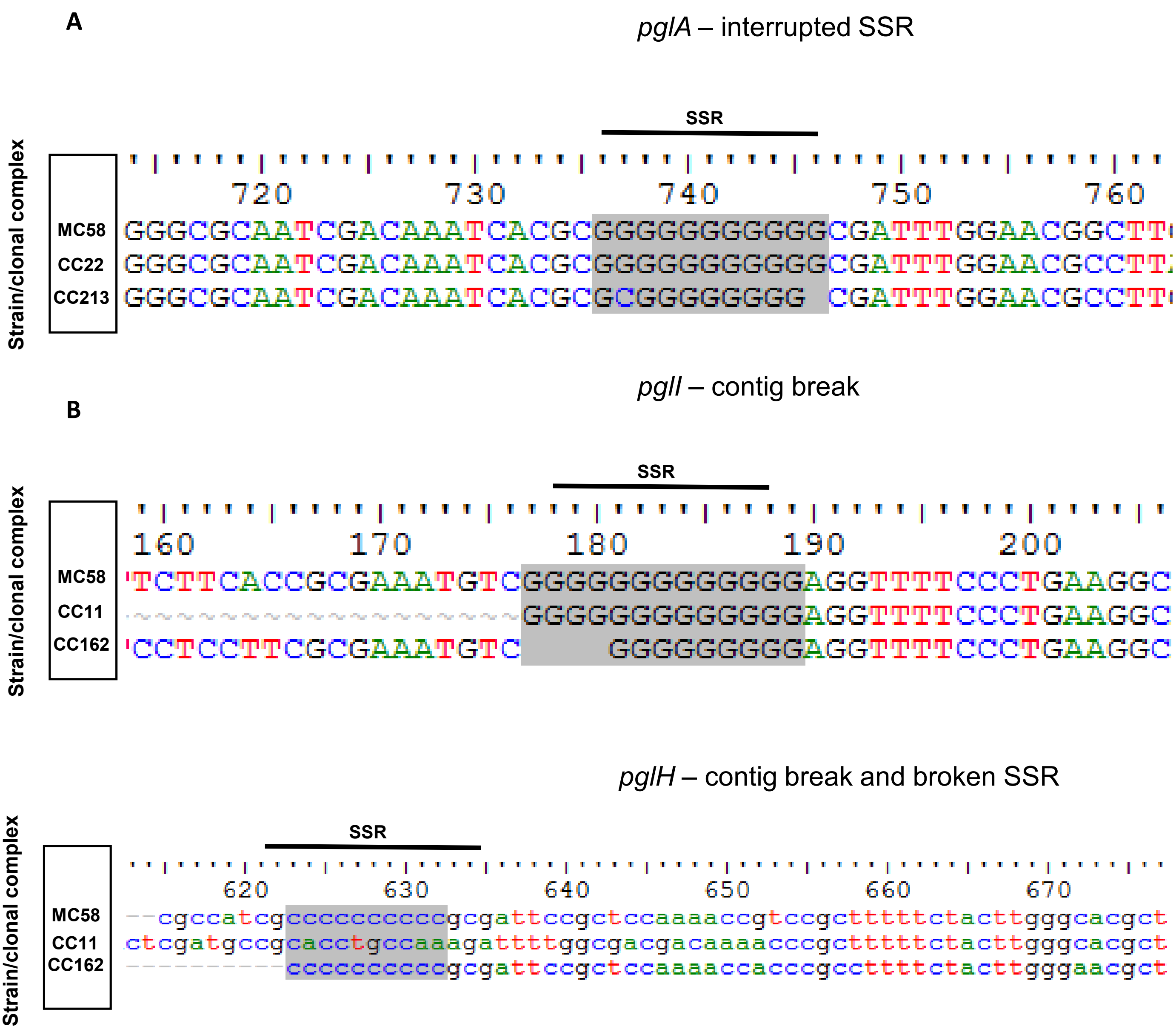

Supplementary Figure 7. Sequence alignments indicating the origin of SSRs at the *pgl* locus. (A) nucleotide sequence alignment of the polyG SSR from *pglA* from the reference strain MC58, a representative cc22 strain, and a representative cc213 strain. (B) nucleotide sequence alignment of the polyG SSR from *pglI* from the reference strain MC58, a representative cc11 strain, and a representative cc162 strain. (C) nucleotide sequence alignment of the polyG SSR from *pglH* from the reference strain MC58, a representative cc22 strain, and a representative cc213 strain.
