# Supplementary material for: Meningococcal core and accessory phasomes vary by clonal complex
Source: Microb Genom. 2020 Apr 29;6(5):e000367. doi: 10.1099/mgen.0.000367 (PMC7371114; doi:10.1099/mgen.0.000367)
Supplement: Supplementary material 3 [file mgen-6-367-s003.pdf]

Tukey's multiple comparisons test	Mean Difference	95.00% Confidence Interval of Difference.	Adjusted P Value
cc11 vs. cc162	-6.914	-8.426 to -5.401	<0.0001
cc11 vs. cc213	-0.7793	-1.689 to 0.1307	0.1694
cc11 vs. cc22	-0.2963	-1.718 to 1.125	0.9997
cc11 vs. cc23	2.827	2.141 to 3.513	<0.0001
cc11 vs. cc269	-0.03861	-0.7121 to 0.6349	>0.9999
cc11 vs. cc32	-2.89	-3.92 to -1.86	<0.0001
cc11 vs. cc41/44	2.01	1.381 to 2.639	<0.0001
cc11 vs. cc461	-0.1289	-1.585 to 1.327	>0.9999
cc11 vs. New ST	-0.7548	-1.683 to 0.1735	0.2303
cc162 vs. cc213	6.135	4.474 to 7.795	<0.0001
cc162 vs. cc22	6.618	4.63 to 8.605	<0.0001
cc162 vs. cc23	9.741	8.191 to 11.29	<0.0001
cc162 vs. cc269	6.875	5.331 to 8.419	<0.0001
cc162 vs. cc32	4.024	2.294 to 5.753	<0.0001
cc162 vs. cc41/44	8.924	7.398 to 10.45	<0.0001
cc162 vs. cc461	6.785	4.773 to 8.797	<0.0001
cc162 vs. New ST	6.159	4.488 to 7.83	<0.0001
cc213 vs. cc22	0.483	-1.096 to 2.062	0.9939
cc213 vs. cc23	3.606	2.636 to 4.576	<0.0001
cc213 vs. cc269	0.7407	-0.2209 to 1.702	0.3031
cc213 vs. cc32	-2.111	-3.348 to -0.8732	<0.0001
cc213 vs. cc41/44	2.789	1.858 to 3.72	<0.0001
cc213 vs. cc461	0.6504	-0.9589 to 2.26	0.9583
cc213 vs. New ST	0.0245	-1.13 to 1.179	>0.9999
cc22 vs. cc23	3.123	1.663 to 4.584	<0.0001
cc22 vs. cc269	0.2577	-1.197 to 1.713	>0.9999
cc22 vs. cc32	-2.594	-4.244 to -0.9433	<0.0001
cc22 vs. cc41/44	2.306	0.8711 to 3.741	<0.0001
cc22 vs. cc461	0.1674	-1.777 to 2.112	>0.9999
cc22 vs. New ST	-0.4585	-2.048 to 1.131	0.9961
cc23 vs. cc269	-2.866	-3.618 to -2.113	<0.0001
cc23 vs. cc32	-5.717	-6.8 to -4.634	<0.0001
cc23 vs. cc41/44	-0.8171	-1.531 to -0.1035	0.0109
cc23 vs. cc461	-2.956	-4.45 to -1.462	<0.0001

cc23 vs. New ST	-3.582	-4.569 to -2.594	<0.0001
cc269 vs. cc32	-2.852	-3.927 to -1.776	<0.0001
cc269 vs. cc41/44	2.048	1.347 to 2.75	<0.0001
cc269 vs. cc461	-0.09031	-1.579 to 1.398	>0.9999
cc269 vs. New ST	-0.7162	-1.695 to 0.2627	0.3785
cc32 vs. cc41/44	4.9	3.851 to 5.949	<0.0001
cc32 vs. cc461	2.761	1.081 to 4.441	<0.0001
cc32 vs. New ST	2.135	0.8842 to 3.386	<0.0001
cc41/44 vs. cc461	-2.139	-3.608 to -0.6698	0.0002
cc41/44 vs. New ST	-2.765	-3.714 to -1.816	<0.0001
cc461 vs. New ST	-0.6259	-2.246 to 0.9938	0.9688

Supplementary Table 1. Pairwise, statistical analysis of PV gene frequency in meningococcal ccs. Analysis was performed using a 2-way ANOVA, with multiple comparisons.
